# Varicella-Associated Death of a Vaccinated Child with Leukemia — California, 2012

**Published:** 2014-02-21

**Authors:** Paula Kriner, Karla Lopez, Jessica Leung, Rafael Harpaz, Stephanie R. Bialek

**Affiliations:** 1 Imperial County Public Health Department; 2 Division of Viral Diseases, National Center for Immunization and Respiratory Diseases, CDC

Varicella, a contagious viral disease, is typically self-limited but can result in serious complications, especially among persons who are immunocompromised (1). On April 10, 2012, a girl aged 4 years with acute lymphoblastic leukemia (ALL) was exposed to a mildly ill cousin who developed a varicella rash 2 days later. The episode was reported to the child’s oncologist after 13 days. The girl was prescribed 7 days of oral acyclovir for prophylaxis and concurrently began her scheduled chemotherapy, which included a 5-day course of dexamethasone (prednisone equivalent dose of 23 mg/day). Twenty-two days after her varicella exposure, the girl was taken to an emergency department for fever and abdominal pain. She was treated symptomatically; her caretakers were instructed to discontinue chemotherapy and to follow up with her oncologist. Two days later, the girl returned to the emergency department with a generalized rash. She was hospitalized and treated with intravenous acyclovir and antibiotics. However, she developed multiorgan failure and died on May 7. Varicella was confirmed by polymerase chain reaction testing, and no alternative diagnoses were found for her acute illness.

The patient had received her first dose of varicella vaccine (Varivax) in March 2009. She was diagnosed with ALL in March 2011. At that time, she was varicella-zoster virus (VZV) immunoglobulin G (IgG)-positive.

To date, there have been five deaths, including this death, reported to CDC among U.S. children who had received 1 dose of varicella vaccine. Four of these deaths occurred among children being treated with immunosuppressive medications; high-dose corticosteroids were a component of their treatments. This patient’s fatal varicella likely was the result of profound immunosuppression, resulting in part from the chemotherapy and corticosteroid treatment (2).

At the time of her ALL diagnosis, this patient had evidence of immunity to varicella (1) based on detection of VZV IgG; postexposure treatment with varicella zoster immune globulin (VariZIG) was not indicated by existing Advisory Committee on Immunization Practices (ACIP) recommendations (3). However, detection of VZV IgG after 1 dose of varicella vaccine might not correspond to adequate protection in immunocompromised persons (1). Because of challenges in assessing protection against varicella in immunocompromised patients, postexposure VariZIG for selected VZV-seropositive persons, such as hematopoietic-cell transplantation recipients, has been recommended by some experts, although this is not an ACIP recommendation (4). Clinicians may consider use of postexposure prophylaxis among profoundly immunocompromised patients on an individual basis.

Varicella vaccination has led to significant declines in varicella disease in the United States (1). Eligible persons without evidence of immunity to varicella should receive 2 doses of varicella vaccine (1). Live-attenuated varicella vaccine is contraindicated for immunocompromised persons, but the vaccination program offers protection to these vulnerable persons through herd effects. To provide more targeted herd protection for immunocompromised children, varicella vaccination of their household contacts is recommended (1).
